# Issue Information

**DOI:** 10.1111/acel.12375

**Published:** 2015-07-14

## EDITORS-IN-CHIEF

### Peter Adams

University of Glasgow

CR-UK Beatson Labs

Garscube Estate, Switchback Road,

Bearsden, Glasgow

G61 1BD

United Kingdom

Tel: +(0)141 330 2306

Fax: +(0)141 942 6521

Email: p.adams@beatson.gla.ac.uk

### Adam Antebi

Huffington Center on Aging and Dept of

Molecular and Cellular Biology

Baylor College of Medicine

One Baylor Plaza, Room M-320

Houston, TX 77030 USA

Tel: +1 713 798 6661

Fax: +1 713 798 4161

Email: aantebi@bcm.tmc.edu

Max Planck Institute for Biology of Ageing

Gleuelerstr. 50A

D-50931 Koeln

Germany

### Ana Maria Cuervo

Depts of Development and Molecular

Biology and of Medicine

Albert Einstein College of Medicine

1300 Morris Park Ave

Bronx, NY 10461 USA

Tel: +1 718 430 2689

Fax: +1 718 430 8975

Email: ana-maria.cuervo@einstein.yu.edu

### Brian Kennedy

President and CEO

Buck Institute for Age Research

8001 Redwood Blvd.

Novato, CA 94945

Tel: 415 209 2040

Fax: 415 899 1810

Email: bkennedy@buckinstitute.org

## REVIEWS EDITOR

**John Sedivy,***Brown University, USA*

Email: john_sedivy@brown.edu

## EDITORIAL BOARD

Genes and functional genomics

*Andrzej Bartke,* S. Illinois Univ., USA

*Lucio Comai,* Univ. of Southern California, USA

*Claudio Franceschi,* Univ. of Bologna, Italy

*David Gems,* UCL, UK

*Jing-Dong Jackie Han,* Chinese Academy of Sciences, China

*Matt Kaeberlein,* Univ. of Washington, USA

*Cynthia Kenyon,* UCSF, USA

*Siu Sylvia Lee,* Cornell Univ., USA

*Valter Longo,* Univ. of S. California, USA

*Colleen T Murphy,* Princeton Univ., USA

*Sang Chul Park,* Well Aging Research Center, Korea

*Linda Partridge,* UCL, UK

*Marc Tatar,* Brown Univ., USA

*Stefan Schreiber,* Christian-Albrechts-Universität, Germany

*Xiao-Li Tian,* Institute of Molecular Medicine, Peking Univ., China

*Jan Vijg,* Albert Einstein College of Medicine, USA

Epigenetics

*Shelley Berger,* Univ. of Pennsylvania, USA

*Anne Brunet,* Stanford Univ., USA

*Mario F Fraga,* CNB/CSIC and IUOPA, Spain

*Masashi Narita,* Univ. of Cambridge, USA

*Cell proliferation, senescence and* death

*Fabrizio d’Adda di Fagagna,* FIRC Institute of Molecular Oncology, Italy

*Maria Blasco,* Spanish Nat. Cancer Center, Spain

*Judith Campisi,* Lawrence Berkeley Nat. Laboratory, USA

*Sandy Chang,* MD Anderson Cancer Center, USA

*Yosef Gruenbaum,* the Hebrew Univ. of Jerusalem, Israel

*Thorsten Hoppe,* Univ. of Cologne, Germany

*Peter Hornsby,* UTHSC at San Antonio, USA

*Eun Seong Hwang,* Univ. of Seoul, Korea

*Pidder Jansen-Dürr,* Inst. for Biomed. Aging Res., Austria

*Brad Johnson,* Univ. of Pennsylvania, USA

*Jan Karlseder,* Salk Inst. for Biological Studies, USA

*Joao Passos,* Newcastle Univ., UK

*Manuel Serrano,* Spanish Nat. Cancer Res. Center, Spain

*Thomas von Zglinicki,* Univ. of Newcastle, UK

Signaling and gene expression

*Johan Auwerx,* Ecole Polytechnique Fédérale, Switzerland

*T Keith Blackwell,* Joslin Diabetes Center, USA

*Marcia Haigis,* Harvard Medical School, USA

*Shin-ichiro Imai,* Washington Univ., USA

*Pankaj Kapahi,* Buck Institute, USA

*David Lombard,* Univ. of Michigan, USA

*Jeffrey S Smith,* University of Virginia, USA

*Giulio Taglialatela,* Univ. of Texas Medical Branch, USA

Cell stress and damage

*Martin Brand,* MRC Dunn Human Nutrition Unit, UK

*Donato Di Monte,* German Center for Neurodegenrative Diseases, Germany

*Deborah A Ferrington,* Univ. of Minnesota, USA

*Toren Finkel,* NIH, USA

*Judith Frydman,* Stanford Univ., USA

*Brad Gibson,* Buck Institute for Age Research, USA

*Robert Goldman,* Northwestern Univ., USA

*Ming Guo,* Univ. of California, USA

*Malcolm Jackson,* Univ. of Liverpool, UK

*Ursula Jakob,* Univ. of Michigan, USA

*Nils-Göran Larsson,* Max Planck Institute for Biology of Ageing, Germany

*Richard A Miller,* Univ. of Michigan Dept of Pathology and Geriatrics Center

*Vincent Monnier,* Case Western Reserve Univ., USA

*Peter Rabinovitch,* Univ. of Washington, USA

*Gerald S Shadel,* Yale University School of Medicine, USA

*Holly Van Remmen,* Oklahoma Medical Research Foundation, USA

Stem cells and aging

*Victoria Ballard,* GlaxoSmithKline, USA

*Mark R Cookson,* National Institute on Aging, USA

*Jay Edelberg,* Cornell Univ., USA

*Heinrich Jasper,* Buck Insititute, USA

*Thomas Nystrom,* Goteborg Univ., Sweden

*Bradley Olwin,* Univ. of Colorado, USA

*Charlotte Peterson,* Univ. of Kentucky, USA

*Thomas A Rando,* Stanford Univ., USA

*Clifford J Rosen,* Tufts Univ. School of Medicine, USA

*Ashok Shetty,* Inst. for Regenerative Medicine at Texas A&M Health Science Center and Scott & White, Temple, TX

Immunology

*Bonnie Blomberg,* Univ. of Miami Miller, School of Medicine, USA

*Daniel Goldstein,* Yale Univ., USA

*Laura Haynes,* Trudeau Inst., USA

*Janet M Lord,* MRC Centre for Immune Regulation, UK

*Janko Nikolich-Zugich,* Univ. of Arizona, USA

*Susan Swain,* Univ. of Massachusetts, USA

*Joanne Turner,* Ohio State Univ., USA

Integrative physiology

*Julie K Andersen,* Buck Inst. for Age Research, USA

*Steven J. Burden,* New York Univ., USA

*Qian Chen,* Brown Univ., USA

*Cheryl Conover,* Mayo Clinic, USA

*Rafael De Cabo,* Nat. Inst. on Aging, USA

*Luigi Fontana,* Washington Univ., USA & Salerno Univ., Italy

*Laura Haynes,* Tudeau Inst., USA

*James Kirkland,* Mayo Clinic, USA

*Simin Meydani,* Tufts Univ., USA

*Ralph Nixon,* NYU School of Med., USA

*Henry L Paulson,* Univ. of Michigan, USA

*Brun Ulfhake,* Karolinska Inst., Sweden

*Jean Wei,* Univ. of Arkansas, USA

*Biodemography and comparative* studies

*Richard M Cawthon,* Univ. of Utah, USA

*Kaare Christensen,* Univ. of S. Denmark, Denmark

*Vera Gorbunova,* Univ. of Rochester, USA

*Nicola Neretti,* Brown University, USA

*Scott Pletcher,* Huffi ngton Center of Aging, USA

*Eline Slagboom,* Univ. of Leiden, Netherlands

*Yousin Suh,* Albert Einstein College of Medicine, USA

Theories of aging and longevity

*Steven Austad,* Univ. of Texas, USA

*Tom Kirkwood,* Univ. of Newcastle, UK

*Daniel Promislow,* University of Georgia, USA

## SCOPE

The aim of *Aging Cell* is to publish the highest quality, innovative research addressing fundamental issues in the biology of aging. For publication in *Aging Cell*, the work must provide a major new contribution to the understanding of aging and be of general interest to the community. *Aging Cell* seeks to cover all areas of geroscience, highlighting research that uncovers mechanistic aspects of the aging process, as well as the links between aging and age-related disease. Observations of novel aging processes without substantial mechanistic insight will be considered, but should be of especially high impact for the field. Topics including, but not limited to, nutrient-responsive signaling pathways, neuronal and endocrine signaling pathways, tissue interactions, genetic and epigenetic regulation and integrity, proteostasis, circadian rhythms, ROS and mitochondria, cellular senescence, stem cells, progerias and interventions that affect aging are encouraged. All experimental approaches, including plant models, are welcome. Papers that focus on the pathogenesis of a specific age-related pathology are also of interest, but should offer new insights into the fundamental links between aging and disease.

****Open Access and Copyright****. All articles published by Aging Cell are fully open access: immediately freely available to read, download and share. All Aging Cell articles are published under the terms of the Creative Commons Attribution License which permits use, distribution and reproduction in any medium, provided the original work is properly cited, and allows the commercial use of published articles.

Copyright on any research article in a journal published by Aging Cell is retained by the author(s). Authors grant Wiley a license to publish the article and identify itself as the original publisher. Authors also grant any third party the right to use the article freely as long as its integrity is maintained and its original authors, citation details and publisher are identified. Further information about open access license and copyright can be found on http://www.wileyopenaccess.com/details/content/12f25db4c87/Copyright--License.html.

****Use by commercial ‘for-profi t’ organisations****. Use of Wiley Open Access articles for commercial, promotional, or marketing purposes requires further explicit permission from Wiley (http://corporatesales@wiley.com) and will be subject to a fee. Commercial purposes include:

- Copying or downloading of articles, or linking to such articles for further redistribution, sale or licensing;
- Copying, downloading or posting by a site or service that incorporates advertising with such content;
- The inclusion or incorporation of article content in other works or services (other than normal quotations with an appropriate citation) that is then available for sale or licensing, for a fee (for example, a compilation produced for marketing purposes, inclusion in a salespack);
- Use of article content (other than normal quotations with appropriate citation) by forprofi t organisations for promotional purposes;
- Linking to article content in e-mails redistributed for promotional, marketing or educational purposes;
- Use for the purposes of monetary reward by means of sale, resale, licence, loan, transfer or other form of commercial exploitation such as marketing products;
- Print reprints of Wiley Open Access articles can be purchased from http://corporatesales@wiley.com.

## Disclaimer

The Publisher, the Anatomical Society and Editors cannot be held responsible for errors or any consequences arising from the use of information contained in this journal; the views and opinions expressed do not necessarily reflect those of the Publisher, the Anatomical Society and Editors; neither does the publication of advertisements constitute any endorsement by the Publisher, the Anatomical Society and Editors of the products advertised.

Wiley Open Access articles posted to repositories or websites are without warranty from Wiley of any kind, either express or implied, including, but not limited to, warranties of merchantability, fitness for a particular purpose, or non-infringement. To the fullest extent permitted by law Wiley disclaims all liability for any loss or damage arising out of, or in connection with, the use of or inability to use the content.

